# Supplementary material for: ‘In search of lost time’: Identifying the causative role of cumulative competition load and competition time-loss in professional tennis using a structural nested mean model
Source: PLoS One. 2020 Apr 17;15(4):e0231568. doi: 10.1371/journal.pone.0231568 (PMC7164605; doi:10.1371/journal.pone.0231568)
Supplement: S1 File — (PDF) [file pone.0231568.s001.pdf]

Supplementary material for 'In Search of Lost Time: Identifying the causative role of cumulative competition load and competition time-loss in professional tennis using a structural nested mean model'

Causes of Time-Loss

Understanding what incentivizes players to compete begins with a review of the structure of professional tennis. The modern-day sport is comprised of four global sports organizations: the Grand Slam Committee, the Association of Tennis Professionals World Tour, the Women's Tennis Association, and the International Tennis Federation. These organizations are responsible for creating opportunities for tennis players to compete and make earnings through prize money won [1]. Professional tennis players are not salaried professionals. Their earnings are based on their appearance and performance at tour events.

In addition to the opportunity for prize money, the sport's organizations incentives players to compete by creating bonus pools of prize money for performance over multiple events, securing ranking points (the basis of eligibility and seeding at events), and penalties for withdraws, which are most severe for higher ranked players. Although less public information is available about the amount and frequency of appearance fees and endorsement deals, it can be assumed that these are additional strong incentives for top players to compete regularly at a high level [2, 3].

Given the economic incentives outlined above, the reasons why a professional tennis player who is making their living from competition would choose not to compete in a particular week are few in number. Indeed the reasons can be fit into six broad categories.

First, a player will be out-of-competition when it is the 'off-season'. The notion of 'off-season' is quoted in this instance because there is no official off-season in tennis and the exact amount of time that a player takes off each year is their choice. However, the calendar for top players has few events in November and December. In 2018, for example, there was only 1 event during this time. This makes November and December a de facto break period for top players. At lower level events, it is possible for players to play throughout the year. However, at this level of the sport, events are smaller and the competitive pool is much larger so the number of play opportunities to the number of potential players is very imbalanced.

Second, a player may not compete if they were unable to gain entry to their preferred event. Eligibility is determined by a player's offical ranking[1]. A highly-ranked player may earn a spot in the event as a direct acceptance. Lower-ranked players will usually have to qualify, which means being accepted to the qualifying tournament (again, based on ranking) and winning enough matches to

[1] `https://www.atptour.com/en/corporate/rulebook`

earn a qualification spot in the main draw. Because eligibility criteria for events is published well before competition begins, players will know where they are most likely to earn qualifying or direct entry, and we can expect ineligibility to be a rare cause for professionals to miss out on opportunities to compete.

Third, players may choose to take a break from competition outside of the off-season in order to rest or train. Because each week away from competition is a week of missed earnings and possible loss in ranking points, most players will keep this to a minimum. Moreover, it can be expected that players will try to maintain a similar training-competition-rest rhythm from season to season, taking rest periods between events at a similar time and frequency each calendar year. Roger Federer received a lot of attention for his decision to skip the clay seasons in 2017 and 2018, highlighting how unusual a choice like this is for most players [2].

Fourth, players who commit a serious offense against the sport's rules of conduct can suffer bans from competition. These can include drug bans, corruption, or unsportsmanlike conduct. Corruption bans are rare and typically involve lower-ranked players. In 2017, corruption bans issued by the Tennis Integrity Unit in 2018 affected 17 players, only 4 have been top 100 players in their career. Drug bans are also infrequent. In 2018, drug bans were initiated for 5 players including men and women [3].

Fifth, personal issues, such as the loss of a loved one, can cause a player to take an absence from competition. Time-loss in this category can include childbirth. This has happened for some top womens players but is rare, as many women choose not to risk their career by having a child before they have retired [4]. Serena Williams is a recent notable case. Her decision and break from competition to have a child put attention on current rules that are not supportive of this choice.

Being unfit to play is the final major cause of being out-of-competition. Given the structure and incentives of professional tennis and the infrequent causes of other reasons for absence from competition, the predominant cause for time-loss is owing to a drop in physical or mental fitness that requires an extended period of recovery.

Regular Schedules

A broad definition of a professional tennis player is any player who competes at the ITF level or above. This definition includes tens of thousands of players in any given year. The majority of these players are not competing regularly and tournament earnings are not their primary source of income. It is only among the top professional players where regular competition and sustainable earnings are possible [5].

---

[2] https://www.tennisworldusa.org/tennis/news/Roger_Federer/53289/roger-federer-s-fitness-trainer-reveals-reason-behind-skipping-clay/

[3] http://www.tennisintegrityunit.com/investigations-and-sanctions

To determine a reasonable cutoff for a 'regular' professional, we computed the median of the biggest gap between competitive events per player for each calendar month. The summary of the median gap in Figure shows notable variation with the rating of a player at the start of the season, with both low-rated players and very highly-rated players showing more month-to-month variation in their breaks between competition weeks. The horizontal line in this plot marks 21 days. We see that the first rating to have 9 months of the year with median gaps at or below 21 days in length was a player rating of 2300.

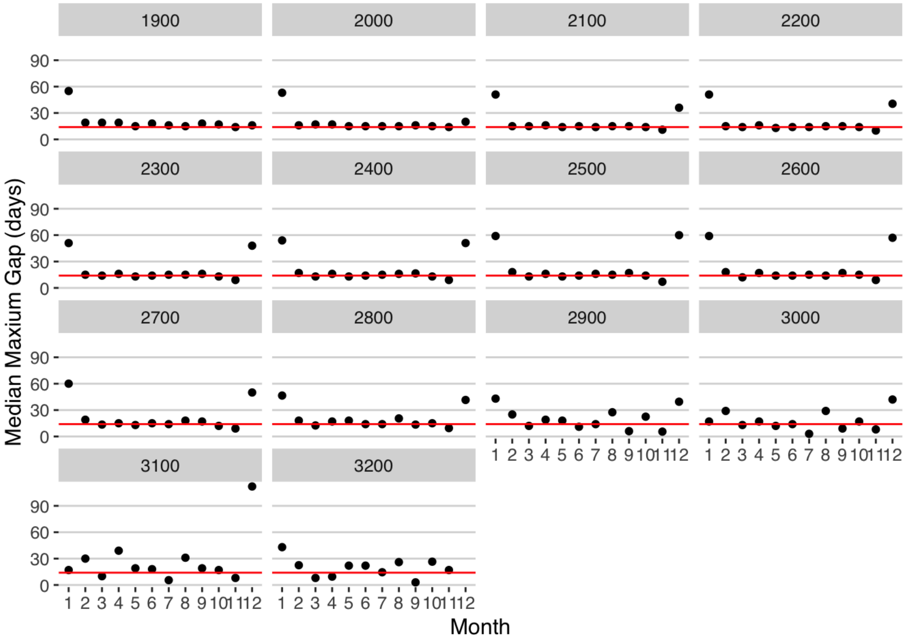

Some of the highest rating levels begin to show greater month-to-month variation, which can be attributed to the small sample of player represented in this extreme end of the ratings distribution.

After limiting schedules to seasons where player's held a rating of 2300 or higher, we can plot each player's maximum gap between competitive events per month to visualize the player-to-player variation (Figure). This reveals the smallest variability in the months of February thru November. However, even for these months, the spread in the maximal days between competition is two weeks for the majority of players.

The months of January and December are of particular interest because of their distinctive patterns in breaks. In January, we can observe two prominent clusters: one with gaps greater than 3 weeks and the other that is less than. These can be explained by players who return to play in December versus January. Among those who return from the off-season in January, the range in

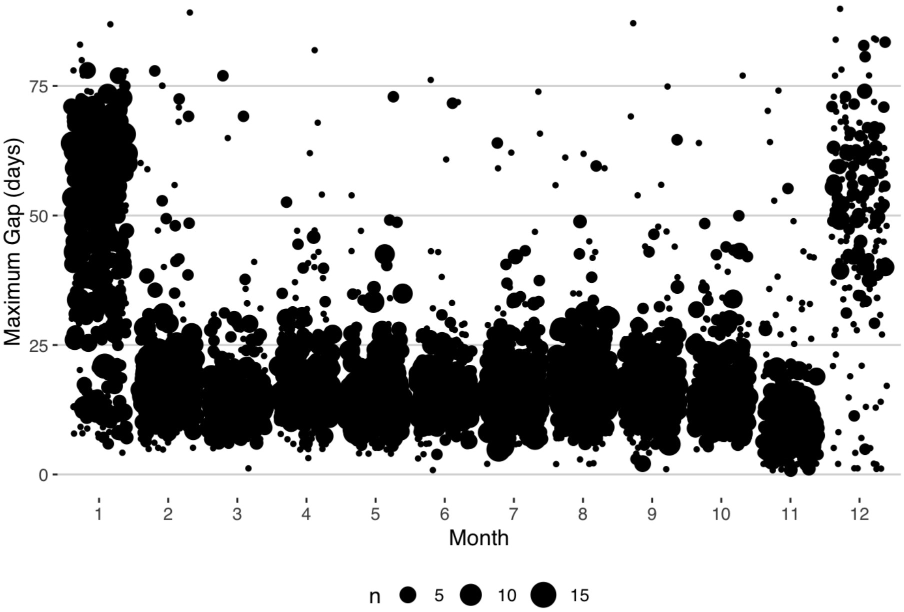

off-season time period is anywhere from 4 to 8 weeks for the majority. A similar but less prominent grouping is observed for the month of December, with most players who return to play in December having returned after a break of 6 to 8 weeks.

Time-Loss Criteria

All player-seasons that began when a player was rated 2300 or higher were used to model the typical days between competitive events. Let D_{ims} be the maximum gap for the ith player, mth month, and s season. The monthly rate for the expected count was modeled as a log-linear mixed effects Poisson,

$$E[log(\lambda|D_{ims})] = \alpha + \beta_m + a_i + b_{im} \quad (1)$$

where a_i is a player specific intercept and b_{im} is the player-specific month effect. The player random effects allow us to obtain conditional expectations for the longest expected gap for each player and month of the season.

The estimated average maximum gap was 14 days. We therefore set the criteria for time-loss to be any gap that was more than 14 days longer than the player's expected maximum gap for the month for all months other than January (the expected return from the off-season) and 28 days longer than the expected gap in January.

Player Validation

Precise records about player injuries are generally not available for professional tennis players. However, some prominent players have detailed accounts of injuries summarized on Wikipedia. These are not expected to be a complete record of all injuries that would meet the definition of a clinical diagnosis but can provide a minimum threshold of injuries that results in extended absences from competition. If an break was significant enough to appear in the press and eventually land on the pages of a players Wikipedia entry, then it should meet the criteria of a time-loss in the present study. We will therefore use the records for a sample of prominent players to assess whether the model-based criteria of time-loss is in good agreement with this subset of injury events. All quoted statements below are taken from each player's respective Wikipedia entry at the time of this writing (June 2019).

The first player we consider is David Nalbandian[4]. Nalbandian is the only Argentine player in history to reach the semifinals or better at all four Grand Slam tournaments and reached the men's singles final at Wimbledon. Nalbandian retired in 2013 after a series of injuries. His Wikipedia entry lists the following extended breaks:

- 'In May, he announced that he would have to undergo a hip surgery, resulting in not being able to compete in the remaining Grand Slams and the Davis Cup [2009]'. A time-loss was identified after Estoril in May of 2009.
- 'The injury persisted, and he withdrew from the 2010 Mutua Madrilea Madrid Open, the 2010 Roland Garros and the 2010 Wimbledon Championships.' A time-loss was identified after Monte Carlo in April of 2010.
- 'Due to a torn hamstring and a hernia, he missed many tournaments including Indian Wells, Miami, Monte Carlo, Madrid and Rome Masters [2011].' A time-loss was identified in March of 2011 following the Davis Cup first round.
- 'Nalbandian withdrew from the 2012 US Open due to a strained muscle in his chest.' A time-loss was identified in August of 2012 after Nalbandian competed in Winston Salem.

The above time-loss events are highlighted among the points that exceeded the gap criteria for Nalbandian (shown by the red lines).

We next consider Andy Murray[5], one of the most successful tennis players in British history.

- 'Murray won both his singles matches, and lost at doubles in the Davis Cup against Poland, but then missed six weeks with a wrist injury [in 2009].'

[4] https://en.wikipedia.org/wiki/David_Nalbandian
[5] https://en.wikipedia.org/wiki/Andy_Murray

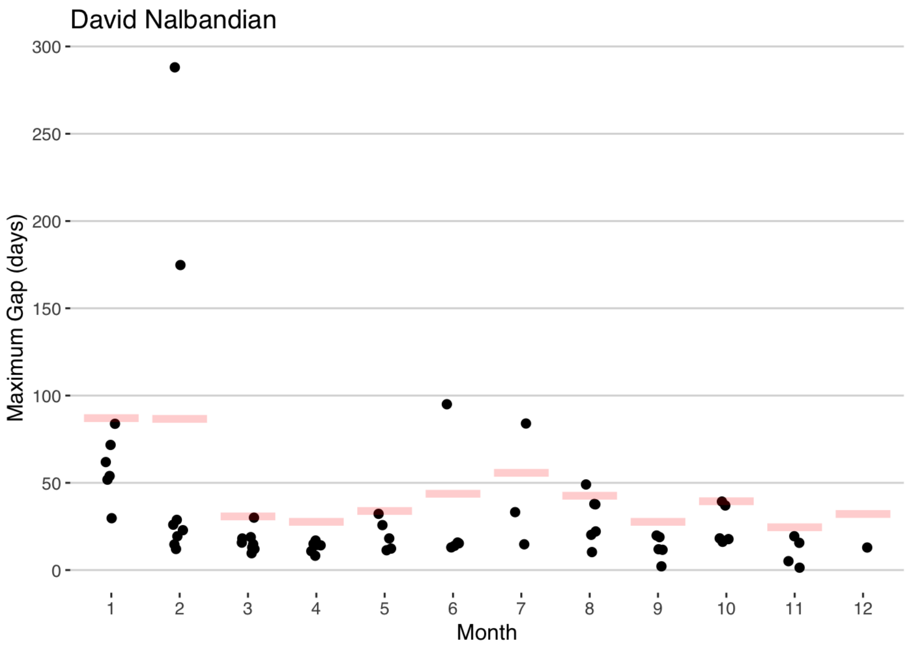

- ‘He would go on to withdraw from Roland Garros later, citing a back injury [in 2013].’ A time-loss was recorded after the Australian Open but the 4-week break during Roland Garros was not inconsistent with gaps in Andy Murray’s schedule at this part of the calendar.
- ‘Murray missed the [2017] Canadian Open and the Cincinnati Masters due to his hip injury, which led to him losing his No. 1 ranking to Rafael Nadal.’ A time-loss was identified following the Indian Wells tournament in March of 2017.
- ‘Murray then withdrew from the Asian hard court swing and said it was ”most likely” that he would not play in a professional tournament again in 2017’. Following, Wimbledon in 2017, a time-loss was identified.
- ‘Murray withdrew from the [2018] Brisbane International and Australian Open due to hip injury’. These missed events were still covered by the break following the 2017 Wimbledon.
- ‘He then announced he would play his first ATP tournament since hip surgery at the Rosmalen Grass Court Championships in June [of 2018’. The time-loss criteria found a significant gap in Murray’s schedule at the end of the 2017 season.
- ‘He withdrew from [the 2018] Wimbledon with a ”heavy heart” a day

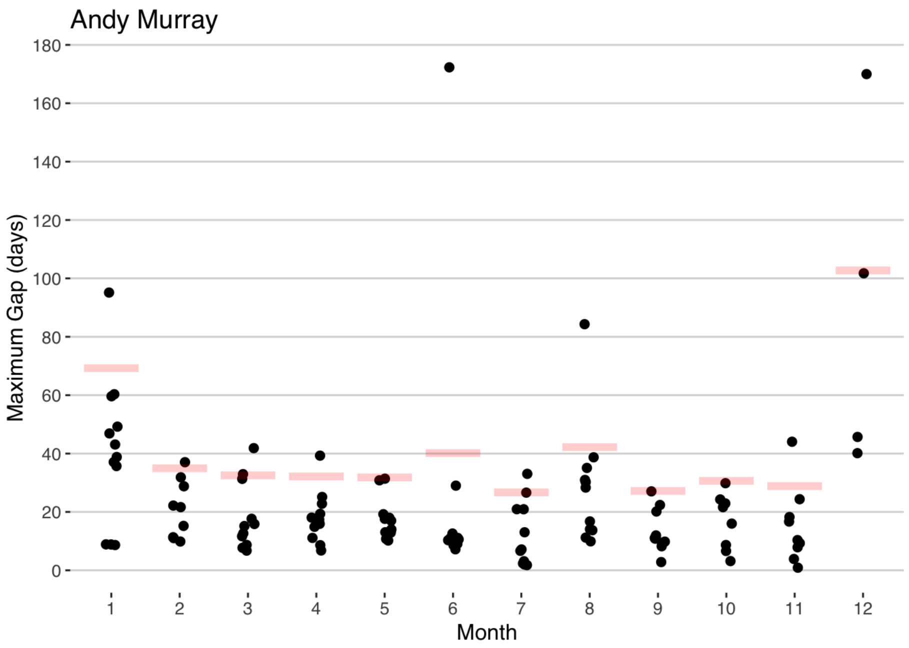

before the tournament, saying it was too soon to play five-set matches'. After returning to Eastbourne in 2018, Murray had a recorded time-loss.

Roger Federer is the most decorated tennis player of the Open Era[6]. In the later part of his career, Federer has also become notable for his longevity, winning multiple Grand Slam titles in his mid-30s. Though, compared to other top tennis player, Federer has stayed healthier for longer, over such a long career he has had several injuries that have forced him to miss competition. The major ones are documented below and compared to the ones identified by the time-loss criteria of the present study.

- 'Near the end of the season he won his hometown tournament, the Swiss Indoors in Basel, Switzerland for the first time, having finished runner up in 2000 and 2001, and missing the tournament in 2004 and 2005 due to injuries'. Time-loss events were identified in October of 2004 and 2005.
- Federer had a time-loss recorded after the 2008 Australian Open, a period when he was diagnosed with mononucleosis[7].
- 'Federer withdrew from the Barclays Dubai Tennis Championships and from Switzerland's Davis Cup tie against the U.S. because of a back in-

[6]https://en.wikipedia.org/wiki/Roger_Federer
[7]https://en.wikipedia.org/wiki/2008_Roger_Federer_tennis_season

jury he sustained in late 2008'. A time-loss was identified after the 2009 Australian Open.

- 'Following the match, Federer was quoted as saying "I was able to play very well. I have to go on holiday badly. I have a problem with my leg, I have a problem with my arm everything is hurting"'. A time-loss was identified after his Davis Cup competition in September of 2009.
- 'Federer withdrew from the Barclays Dubai Tennis Championships for the second consecutive year due to a lung infection [in 2010]'. An extended break was identified after the Australian Open in 2010.
- 'He then continued with his pre-planned decision to take a two-month break from the sport [in 2013], skipping the mandatory Masters 1000 tournament in Miami under an exception from the ATP'. A time-loss was met after Federer's Indian Wells event in March of 2013.
- 'The day after his loss to Djokovic, Federer sustained a knee injury and in early February, he underwent arthroscopic surgery to repair a torn meniscus in his knee and missed the tournaments in Rotterdam, Dubai and Indian Wells in February and March [of 2016].' Federer had a length gap following the Australian Open in 2016 that was flagged as a time-loss.
- 'In Madrid [in 2016], he suffered a back injury during practice and withdrew shortly after arriving'. A second time-loss in 2016 occurred following Wimbledon.
- 'Due to concerns about his longevity, Federer decided that he would skip the entire clay-court season [in 2017]'. Federer's decision to skip the clay court seasons in 2017 and 2018 triggered time-loss events in both years.

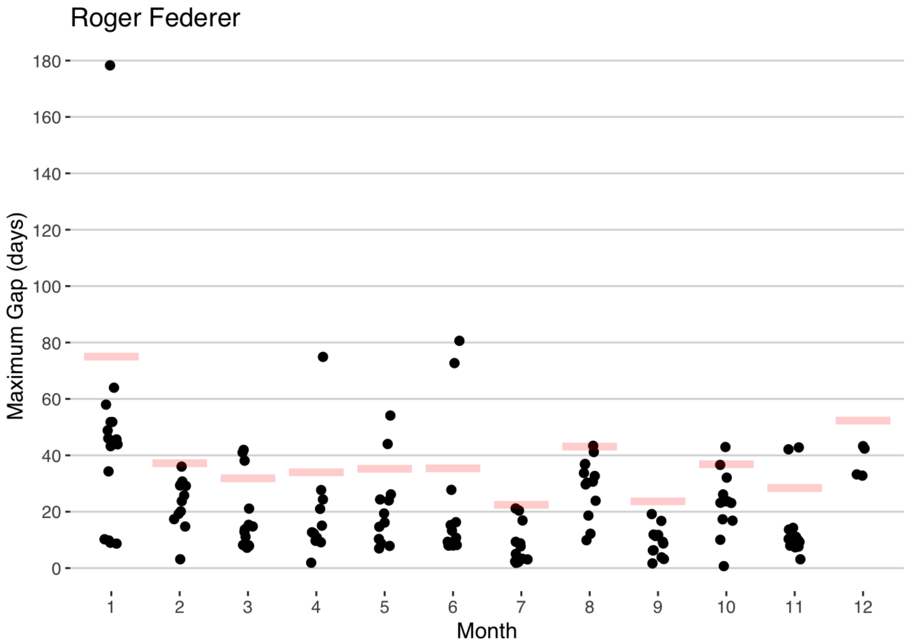
Roger Federer
180
160
140
120
100
80
60
40
20
0
Maximum Gap (days)
1
2
3
4
5
6
7
8
9
10
11
12
Month

References

[1] Gibson AD. The Association of Tennis Professionals: From Player Association to Governing Body. Journal of Applied Business & Economics. 2010;10(5).

[2] Du Bois C, Heyndels B. Labour supply decisions of professional tennis players: Determinants of tournament entry. European Sport Management Quarterly. 2009;9(3):333–344.

[3] Geyer H. Quit behavior of professional tennis players. Journal of Sports Economics. 2010;11(1):89–99.

[4] McGannon KR, McMahon J, Schinke RJ, Gonsalves CA. Understanding athlete mother transition in cultural context: A media analysis of Kim Clijsters tennis comeback and self-identity implications. Sport, Exercise, and Performance Psychology. 2017;6(1):20.

[5] von Allmen P. The Economics of Individual Sports: Golf, Tennis, Track, and NASCAR. In: Handbook of sports economics research. Routledge; 2017. p. 149–171.
